# One-Stage Posterior Debridement and Transpedicular Screw Fixation for Treating Monosegmental Thoracic and Lumbar Spinal Tuberculosis in Adults

**DOI:** 10.1155/2014/137106

**Published:** 2014-02-19

**Authors:** Zhili Liu, Jiaming Liu, Aifeng Peng, Xinhua Long, Dong Yang, Shanhu Huang

**Affiliations:** ^1^Department of Orthopedic Surgery, The First Affiliated Hospital of Nanchang University, No. 17 Yongwaizheng Street, Donghu District, Jiangxi Province, Nanchang 330006, China; ^2^Department of Orthopedic Surgery, Jiangxi University of Traditional Chinese Medicine, Nanchang 330006, China

## Abstract

Spinal tuberculosis is still prevalent in some developing countries. The purpose of this study is to investigate the efficacy and safety of one-stage posterior debridement, autogenous bone grafting, and transpedicular screw fixation in treating monosegmental thoracic and lumbar tuberculosis in adults. 37 patients were retrospectively reviewed in this study. The data of images, operative time and blood loss volume, perioperative complications, time to achieve bony fusion, VAS score, and neurologic function preoperatively and postoperatively were collected. The mean follow-up period was 21.5 ± 3.5 months. The tuberculosis was cured after surgery in all patients, and no recurrence was observed. Bony fusion was achieved in all patients with a mean time of 5.6 ± 2.5 months. Neurological outcome did not change in one case with grade A, and increased by 1–3 grades in the other patients with nerve deficit. The average preoperative and postoperative VAS scores were 5.5 ± 2.23 and 1.5 ± 1.22, respectively; the difference was significant (*P* < 0.05). There were three perioperative complications (8.1%, 3/37) observed in this study. In conclusion, the procedure of one-stage posterior debridement, interbody fusion with autogenous bone grafting, and posterior fixation with pedicle screw is effective and safe for treating monosegmental thoracic and lumbar spinal tuberculosis in adults.

## 1. Introduction

Spinal tuberculosis (TB) is a destructive form of tuberculosis. It accounts for approximately half of all cases of musculoskeletal tuberculosis. The incidence of spinal tuberculosis is increasing in developing countries. Gross destruction of the vertebral bodies and disc by tuberculosis often leads to kyphotic deformity [1, 2]. Though many patients can be cured by chemotherapy, surgery is frequently imperative for spinal decompression and deformity correction [3]. Previous studies [4, 5] showed that surgical treatment is an important strategy for the treatment of spinal TB.

In 1934, Ito et al. firstly described anterior approach for spinal TB. However, anterior debridement may reduce the biomechanical stability of the spine and it is common to find residual kyphosis at the end of treatment [6]. Thus, anterior debridement combined with posterior fusion and fixation was developed, which helped to arrest the disease early, provide early fusion, prevent the progression of kyphosis, and also correct it [7–10]. However, the combined procedures were associated with longer operating time, greater blood loss, more postoperative complications, and longer hospital stay [11, 12]. The purpose of the present study is to investigate the efficacy and safety of one-stage posterior debridement, autogenous bone grafting, and transpedicular screw fixation in treating monosegmental thoracic and lumbar spinal tuberculosis in adults.

## 2. Materials and Methods

This study was approved by the ethics board committee of our hospital, and informed consents were obtained from all participants. The clinical data of thirty-seven consecutive patients with monosegmental thoracic and lumbar spinal tuberculosis, who were treated with one-stage posterior debridement, transpedicular fixation, and fusion with autogenous bone grafting at the First Affiliated Hospital of Nanchang University between January 2010 and June 2011, were retrospectively analyzed. 21 of the patients were males and 16 were females, aged from 22 to 68 years old (average 42.5 years) at the time of surgery. The diagnosis of spinal tuberculosis was guided by symptoms, by laboratory findings such as anemia, hypoproteinemia, erythrocyte sedimentation rate (ESR), and C-reactive protein (CRP), and by imaging including spinal X-ray films, computed tomography, and magnetic resonance imaging. All the patients presented with constitutional symptoms including weakness, night sweats, back pain and stiffness, local tenderness, and lower fever with weight loss. Preoperative computed tomography (CT) and/or magnetic resonance imaging (MRI) examinations showed monosegment vertebral bone destruction, uneven signals of bone, smaller intervertebral space, paravertebral abscess, lack of sinus tract formation, and paraspinal abscess formation. Also, the patients were identified as spinal TB by histopathological evaluation. The patients whose spinal lesions were confined to monosegment with/without neurology and/or deformity formation were involved in this study. Patients with two or more segments spinal tuberculosis were excluded from this study.

Four patients had been previously treated for pulmonary tuberculosis. Diabetes mellitus was identified in 6 patients. 13 patients were active cigarette smokers. Five cases with a psoas muscles abscess, 9 cases with a deep abscess in the vertebral canal and a compressed dural sac were included. Damage to the vertebra was as follows: 1 case with T3-4 damage, 2 cases with T5-6 damage, 4 cases with T8-9 damage, 5 cases with T10-11 damage, 9 cases with T11-12 damage, 11 cases with T12-L1 damage, 3 cases with L2-3 damage, and 2 cases with L3-4 damage.

### 2.1. Preoperative Management

When a clinical diagnosis of spinal TB was made and active pulmonary tuberculosis was excluded, patients were treated with four first-line antitubercular drugs (rifampicin, isoniazid, streptomycin, and pyrazinamide) for at least two weeks before surgery. The operation was performed when ESR and CRP significantly decreased, TB toxicity symptoms obviously improved, and nutritional state got better.

### 2.2. Operative Procedure

The patients were placed in prone position after administration of general anesthesia. A linear midline skin incision of the back was done. Then, posterior elements including lamina, facet joints, and transverse processes were exposed. The pedicle screw system was installed to stabilize and correct the kyphosis in all patients, at least two levels superior and inferior to the level of decompression. If the upper part of the vertebral body was not destroyed by TB, the affected vertebrae were often incorporated into the pedicle screws. Then, a temporary rod on the mild side of the focus was stabilized to avoid spinal cord injury caused by instability of the spine during decompression and focal debridement. The spinous process, bilateral or unilateral lamina, facet joints, costotransverse articulations, and medial 4.0–6.0 cm of rib at the level of decompression were resected for debridement (removal of the destroyed vertebral body and disc) and decompression. Thoracic nerve roots on the focal side were sacrificed for better exposure. The temporary rod was switched to another side for complete debridement and decompression. After that, correction of the spinal deformity was accomplished by installing contoured permanent rods with compression maneuvers. When manipulating compression procedure, it should be noticed that the spinal cord, nerve roots, and dural sac were not stretched or distracted. The corticocancellous chips harvested from the local bone and iliac in all patients were implanted into the space created by debridement. The drainage and incision sutures were performed. The debridement pathological tissues were sent for culture and histopathologic examination. No operation was performed to drain the psoas muscles abscess. The operative time, blood loss during surgery, and the perioperative complications were noted.

### 2.3. Postoperative Management

The drainage tubes were usually removed after 48–72 hours (when drainage volume was less than 50 mL/24 h). The patients were allowed to get up and walk with a brace two weeks after the surgery. All patients were treated with an antituberculous chemotherapy regimen for 9–12 months. The usual regimen followed was four-drug chemotherapy (rifampicin, isoniazid, ethambutol, and pyrazinamide) for two months followed by three drugs (rifampicin, isoniazid, and ethambutol) for the remainder of the period.

### 2.4. Follow-Up

Postoperative follow-up visits were done at 1 and 3 months, and interval one month until bony fusion. Subsequent follow-ups were at six-month intervals. At each follow-up evaluation, ESR and CRP were checked. Plain radiographs were obtained in standing positions in order to determine the development or progression of spinal deformity after surgery and instrumentation failure. MRI was carried out to observe the absorption of psoas muscle abscess at first follow-up visit and each six-month interval, until the psoas muscle abscesses were completely absorbed. CT scan and reconstruction were performed for investigating the bony fusion. The lordosis of the surgical level was measured by two radiologists on the lateral plain radiographs in neutral position according to Cobb's method. The clinical and radiologic evidences of bony fusion were defined as the absence of correction loss, instrumentation failure, and the presence of trabecular bone bridging between the bone grafts and the vertebrae. The nerve function and back pain were evaluated by Frankel Scale and Visual Analogue Scale (VAS) score, respectively. Those patients meeting the following criteria were considered to be cured: (1) disappearance of clinical symptoms with the ability to return to normal activities; (2) bony fusion achieved according to radiography; (3) ESR and CRP decrease to normal levels; and (4) no recurrence of spinal tuberculosis appearing one year after surgery.

### 2.5. Statistical Analysis

All measurement data were expressed as $\overset{-}{\text{X}}\pm \text{S}.\text{D}$. The statistical significance of Cobb's angle and VAS score before and after surgery and at the last follow-up were assessed using one-way ANOVA test. The Chi-square test was used to evaluate the difference of nerve function pre- and postoperation. Values of *P* < 0.05 were considered to be significant differences. All analyses were performed using SPSS Version 13.0 (Statistical Software for Social Sciences, Chicago, IL).

## 3. Results

### 3.1. Laboratory Data

Patients were followed up for at least twelve months, with an average of 21.5 ± 3.5 months (range 12–28 months). The average preoperative ESR and CRP values were 45 ± 15 mm/h (range 25–115 mm/h) and 29 ± 15 mg/L (15–145 mg/L), respectively. They decreased gradually one to two months after the operation and returned to normal three months postoperatively. The spinal tuberculosis was cured at the final follow-up in all the patients, and no recurrence was observed.

### 3.2. Blood Loss and Operation Time

The mean operation time was 192 ± 28 min (range 150–255 min) with an average blood loss volume of 775 ± 394 mL (range 400–1800 mL).

### 3.3. Image Data

**Figure 1:**
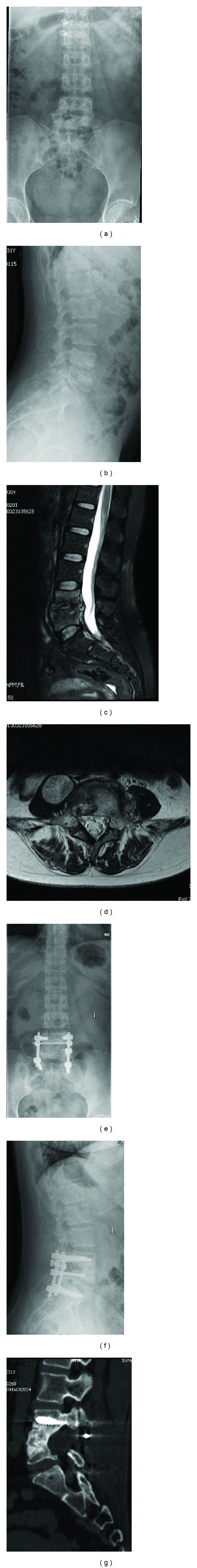
A 30-year-old female patient presented with spinal tubercular spondylodiscitis at L4-5 with a paravertebral abscess (a–d). After one-stage posterior debridement, interbody fusion with autogenous bone grafting, and fixation with pedicle screw, this defect of the spine was reconstructed in sagittal profile (e, f). 12 months postoperatively, a successful bony fusion was observed in reconstruction CT film (g).

**Figure 2:**
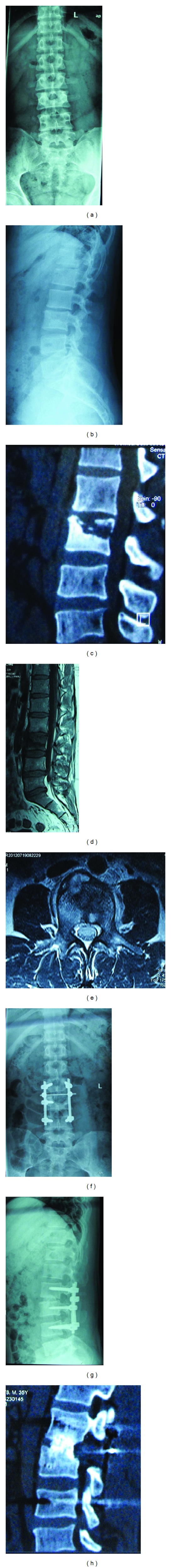
A 33-year-old male patient was diagnosed as having tuberculous spondylitis at L2-3 with a paravertebral abscess after a 2-month history of severe back pain (a–e). After one-stage posterior debridement, interbody fusion with autogenous bone grafting, and fixation with pedicle screw, this focal tuberculosis was removed and the spine was reconstructed in sagittal profile (f, g). 15 months postoperatively, a successful bony fusion was observed in reconstruction CT film (h).

In the patients with psoas muscles abscess, the MRI was performed three months after the surgery, which showed the psoas muscles abscess was absorbed in all of the five patients. All patients presented the evidence of successful bony fusion within a mean time of 5.6 ± 1.5 months (Figures 1 and 2). The average of preoperative and postoperative spinal kyphotic Cobb's angle was 28.3° ± 11.95° and 5.5° ± 11.84°; the difference was significant (*P* < 0.05). Little loss of correction angle was noted in these patients at the final follow-up (7.2° ± 11.56°). However, there was no significant difference between postoperative angle and the final follow-up one (*P* = 0.542).

### 3.4. Perioperative Complications

Three perioperative complications (8.1%, 3/37) were observed in the present study. Two of them suffered from intercostal neuralgia, which was relieved by nonsteroidal anti-inflammatory and neurotrophic drugs. One suffered from the superficial infection around the incision but responded well to early debridement and antibiotics. Deterioration of neurologic status was not noted.

### 3.5. Neurological Status

Neurological outcomes were evaluated by Frankel scale. The results were shown in Table 1. The average preoperative and postoperative VAS scores were 5.5 ± 2.23 (range 1–9) and 1.5 ± 1.22 (range 0–4), respectively; the difference was significant (*P* < 0.05). At the final follow-up, the average VAS score was 0.9 ± 0.88 (range 0–3). There was no significant difference between the postoperative VAS score and the final follow-up visit's score (*P* = 0.075).

## 4. Discussion

Spinal TB is still prevalent in some developing countries. The goals of surgical treatment are focal clearance of TB, relief of spinal nerve compression, and correction of the serious spinal deformity. Certainly, reconstruction of spinal stability is beneficial to cure spinal TB and avoid recurrence. Since Ito et al. firstly introduced anterior approach for spinal TB, anterior debridement combined with posterior fusion and fixation was popular for the treatment of spinal TB. Recently, the trends in surgical treatment of spinal TB have been smaller incisions, static internal fixation, and only one approach during the operation [13, 14]. And one-stage posterior procedure becomes an alternative treatment for spinal TB. Zhang et al. [15] indicated that one-stage posterior approach obtained more satisfactory outcome than posterior plus anterior approach surgery in minor surgical invasion and less procedure-related complications.

In the present study, we performed the procedure of one-stage posterior debridement, fixation with pedicle screw, and corticocancellous chips grafting for the treatment of 37 patients with monosegmental thoracic and lumbar spinal TB. The mean operation time was 192 min with an average blood loss of 775 mL. The operation time and blood loss were shorter than those reported by Pu et al. [16]. In Pu et al.'s study, the mean duration of surgery was 390.2 min and average blood loss was 834.1 mL. Three patients (8.1%, 3/37) presented slight perioperative complications in the present study and all recovered within three months, which did not affect the bony fusion of the spine.

Ma et al. [17] reported that excellent neurological result was observed after single stage posterior debridement, bone graft, and internal fixation in patients with neurological impairment due to spinal TB, which was similar to those obtained via anterior decompression. And the posterior approach may be superior to the anterior instrumentation to correct deformity and maintain that correction. In the present study, the Frankel scores were significantly higher at the final follow-up visit than those before surgery (*P* < 0.05). The results were consistent with Zhang et al.'s study [18]. The VAS scores of the patients were decreased from 5.5 before surgery to 0.9 at the final follow-up visit. The ESR and CRP significantly decreased within three months postoperatively. The bony fusion was achieved in all patients within a mean time of 5.6 months, which was shorter than that reported by Zhang et al. [15]. In Zhang et al.'s study, the interbody fusion occurred averagely 8.3 months after the surgery. The preoperative and postoperative mean kyphotic Cobb's angle were 28.3° ± 11.95° and 5.5 ± 11.84° in the present study, and the difference was significant (*P* < 0.05). No significant loss of deformity correction was noted in these patients at the final follow-up (*P* = 0.542). The result was similar to Zhang et al.'s study [19]. It was concluded from our study that the final outcome of one-stage posterior approach was satisfying.

There are still some limits for this technique due to the authors' experience and insufficient sample of patients. Firstly, posterior debridement bears the potential risk of TB spreading to the posterior healthy regions, resulting in infection diffusion and fistulas. Fortunately, these complications have not been observed in the present study. Secondly, the normal posterior column of spine was destroyed to achieve complete debridement and decompression in this procedure, which would affect the stability of the spine in theory. Long-term follow-up is needed to observe the stability of the spine.

In conclusion, the procedure of one-stage posterior debridement, transpedicular screw fixation, and fusion with autogenous bone grafting is an effective and safe procedure for surgical treatment of the monosegmental thoracic and lumbar spinal TB in adults. Although the results from this study showed that the kyphosis was corrected and maintained better at the final follow-up, this is only a short-term follow-up. Further study with a large number of patients and longer follow-up will be necessary.

## Figures and Tables

**Table 1:** The preoperative and final follow-up of neurological function was evaluated by Frankel scale.

Frankle grade					
	A	B	C	D	E
Preoperative	3	5	11	9	9
Postoperative	1	2	4	9	21

*χ* ^2^ value	29.2				
*P* value	0.000				
